# Oxidative Stress-Induced Damage to the Developing Hippocampus Is Mediated by GSK3β

**DOI:** 10.1523/JNEUROSCI.2389-21.2022

**Published:** 2022-06-15

**Authors:** Joseph Abbah, Claire-Marie Vacher, Evan Z. Goldstein, Zhen Li, Srikanya Kundu, Brooke Talbot, Surajit Bhattacharya, Kazue Hashimoto-Torii, Li Wang, Payal Banerjee, Joseph Scafidi, Nathan A. Smith, Li-Jin Chew, Vittorio Gallo

**Affiliations:** ^1^Center for Neuroscience Research, Children's National Research Institute, Children's National Hospital, Washington, DC 20010; ^2^Bioinformatics Core, Children's National Research Institute, Children's National Hospital, Washington, DC 20010; ^3^Center for Genetic Medicine, Children's National Research Institute, Children's National Hospital, Washington, DC 20010

**Keywords:** Akt, excitation–inhibition balance, GABA, interneurons, memory, POMC

## Abstract

Neonatal brain injury renders the developing brain vulnerable to oxidative stress, leading to cognitive deficit. However, oxidative stress-induced damage to hippocampal circuits and the mechanisms underlying long-term changes in memory and learning are poorly understood. We used high oxygen tension or hyperoxia (HO) in neonatal mice of both sexes to investigate the role of oxidative stress in hippocampal damage. Perinatal HO induces reactive oxygen species and cell death, together with reduced interneuron maturation, inhibitory postsynaptic currents, and dentate progenitor proliferation. Postinjury interneuron stimulation surprisingly improved inhibitory activity and memory tasks, indicating reversibility. With decreased hippocampal levels of Wnt signaling components and somatostatin, HO aberrantly activated glycogen synthase kinase 3 β activity. Pharmacological inhibition or ablation of interneuron glycogen synthase kinase 3 β during HO challenge restored progenitor cell proliferation, interneuron development, inhibitory/excitatory balance, as well as hippocampal-dependent behavior. Biochemical targeting of interneuron function may benefit learning deficits caused by oxidative damage.

**SIGNIFICANCE STATEMENT** Premature infants are especially vulnerable to oxidative stress, as their antioxidant defenses are underdeveloped. Indeed, high oxygen tension is associated with poor neurologic outcomes. Because of its sustained postnatal development and role in learning and memory, the hippocampus is especially vulnerable to oxidative damage in premature infants. However, the role of oxidative stress in the developing hippocampus has yet to be explored. With ever-rising rates of neonatal brain injury and no universally viable approach to maximize functional recovery, a better understanding of the mechanisms underlying neonatal brain injury is needed. Addressing this need, this study uses perinatal hyperoxia to study cognitive deficits, pathophysiology, and molecular mechanisms of oxidative damage in the developing hippocampus.

## Introduction

Numerous preclinical models of neonatal brain injury, such as hypoxia (Scafidi et al., 2009; Titomanlio et al., 2015), infection, placental insufficiency, chorioamnionitis (Chew et al., 2013; Hagberg et al., 2015), and prematurity (Gitto et al., 2009; Iliodromiti et al., 2013; Shevelkin et al., 2014), induce oxidative stress. Thus, oxidative stress may represent a common pathologic mechanism for developmental brain injury, and its underlying molecular mechanisms may offer a viable strategy for intervention. As antioxidant defenses normally develop in the third trimester, preterm infants lack adequate antioxidant capacity (Gerdin et al., 1985; Lázár et al., 2015), raising the risk of oxygen radical diseases (Rogers et al., 2000). High tissue oxygen is associated with poor neurologic outcomes, including cognitive deficit (Collins et al., 2001; Hack et al., 2002; Klinger et al., 2005), but its role in hippocampal damage during development is unexplored.

We hypothesized that premature birth exposes the brain to oxidative stress arising from a twofold to threefold increase in oxygen tension on delivery from the hypoxic uterine environment. To model a significant increase in neonatal oxygen tension, we exposed postnatal day 6 (P6) mice to 80% oxygen [hyperoxia (HO)] for 48 h, a paradigm that produces increased blood partial pressure of oxygen (pO_2_) levels, delayed white matter development, impaired axonal conduction (Schmitz et al., 2011; Ritter et al., 2013), and motor coordination deficits (Schmitz et al., 2012). This HO model, relevant to exposure in the third trimester, produces reactive oxygen species (ROS) in different brain regions (Felderhoff-Mueser et al., 2004; Gerstner et al., 2008; Scheuer et al., 2015). The diffuse nature of HO injury (Schmitz et al., 2011; Ritter et al., 2013) allows for the identification of sensitive neuronal regions that contribute to cognitive impairment. Since the adult hippocampus is a recognized structure of selective vulnerability to oxidative stress (Wang and Michaelis, 2010), we hypothesized that similar vulnerabilities may be found in the developing hippocampus. Studies of neuronal excitation and inhibition show that learning deficits involve synaptic dysregulation in neurodevelopmental disorders (Souchet et al., 2014). Interneurons are known to be critical for functional plasticity (Liguz-Lecznar et al., 2016), which is essential to memory and learning. Based on reports of differential interneuron sensitivity (De la Rosa-Prieto et al., 2016), we hypothesized that developmental oxygen-induced hippocampal damage causes stress-related signaling, which in turn affects cell viability and disrupts signals required for interneuron development.

Glycogen synthase kinase 3 β (GSK3β) is a serine/threonine kinase important in glucose metabolism (Woodgett and Cohen, 1984) and neuronal development. GSK3β levels are highest in the brain, where it controls the development of neural cells under the influence of neurotrophic factors (Hur and Zhou, 2010). However, GSK3β is sensitive to redox homeostasis, and its signaling is involved in oxidative stress-related damage (Wang et al., 2013). In the hippocampus, an appropriate level of GSK3β activity modulates synaptic plasticity involved in memory formation (Peineau et al., 2008). Consequently, both overexpression and deletion of GSK3β alter hippocampus-dependent cognitive functions, such as spatial learning and memory (Hernández et al., 2002; Mao et al., 2009; Liu et al., 2017). Pathologic activation of GSK3β signaling leads to neurodegenerative/neuropsychiatric diseases (Busciglio et al., 1995; Takashima et al., 1996; Ferreira et al., 1997; Mao et al., 2009), but its involvement in oxidative stress-induced neurodevelopmental disorders and cognitive deficits is uncharacterized.

In the present study, we observed that HO increases markers of oxidative stress, decreases proliferation of dentate gyrus (DG) cells, and causes dysmaturation of interneurons to impair GABAergic neurotransmission. HO-induced oxidative stress alters the regulation of GSK3β, resulting in increased levels of the activated form of GSK3β in the hippocampus. We demonstrate that GSK3β activation mediates aberrant hippocampal development caused by HO and that GSK3β inhibition reverses HO-induced cell loss and interneuron dysmaturation, as well as recognition memory. Our results indicate that cognitive impairment resulting from oxidative damage may be alleviated through attenuation of aberrant GSK3β activity.

## Materials and Methods

### Animals.

All animal procedures performed in this study were approved by the Institutional Animal Care and Use Committee of The Children's National Hospital (CNH) in accordance with the National Institutes of Health *Guide for the Care and Use of Laboratory Animals* (protocol #30573). All mouse colonies used in the study were housed in the Comparative Medicine Unit of CNH. The wild-type C57BL/6 (stock #000664), Gad2Cre (stock #010802), Gad2CreER (stock #010702), and POMCCreER (stock #010714) mice were all obtained from The Jackson Laboratory. GAD65-GFP (López-Bendito et al., 2004), proopiomelanocortin (POMC)-EGFP, Gsk3β^flox/flox^, and GCAMP5TdTomato mice were received as donations from Stefano Vicini (Georgetown University, Washington, DC), Gary Westbrook (The Vollum Institute, Oregon Health and Science University, Portland, OR), James Woodgett (Toronto Centre for Phenogenomics, Toronto, ON, Canada), and author N.A.S., respectively. To conditionally delete GSK3β, we crossed Gsk3β^flox/flox^ mice, in which the exon 2 of GSK3β was flanked by loxP sites, with GAD2CreER^T2^/GCAMP5TdTomato and POMCCreER^T2^/GCAMP5TdTomato breeders, and mouse pups were injected with 1 mg of tamoxifen at P4 to remove the STOP sequence and induce recombination, resulting in permanent expression of GCAMP5TdTomato. All transgenic mice used were raised on a C57BL/6 background.

### Hyperoxia.

Hyperoxia was induced in postnatal mice according to the protocol described in Schmitz et al., 2011. P6 pups were randomly assigned into one of two groups: a control group or a hyperoxia group. Control pups from each litter were maintained in room air with a lactating foster dam. Mice in the hyperoxia group, along with a lactating foster dam, were transferred into a transparent plastic chamber maintained with 80–85% of oxygen (O_2_) for 48 h. After exposure, mice were either immediately used for various experimental assays or returned to their home cage and maintained in the room air condition before use at prespecified time points. Our laboratory previously demonstrated this paradigm of hyperoxia exposure to have no significant impact on body weights, metabolic profile, and overall health of pups; pO_2_ was, however, elevated twofold to threefold (Schmitz et al., 2011; Ritter et al., 2013).

### Immunohistochemistry.

Control and HO-exposed mice at different developmental time points were anesthetized with isoflurane and transcardially perfused sequentially with cold 1 m PBS followed by 4% paraformaldehyde (PFA). Brains were collected, transferred into 4% PFA overnight, and sequentially maintained in 10% and 20% glycerol for 24 h, respectively. Serial coronal sections (40 µm thick) of brain sections were obtained using a sliding microtome and stored in PBS containing 0.05% sodium azide maintained at 4°C until use. Floating sections were rinsed in PBS and incubated in a blocking solution [1% bovine serum albumin (BSA), 0.3% Triton X-100, and 20% normal goat serum (NGS) in 1× PBS] at room temperature for 1 h. Sections were incubated in primary antibodies diluted in a carrier solution (1% BSA, 0.3% Triton X-100, and 1% NGS in PBS) overnight at 4°C. The following primary antibodies were used: rabbit anti-sox2 (1:500; Abcam), rat anti-doublecortin (DCX; 1:200; Abcam), chicken anti-GFP (1:200; Abcam), rabbit anti-Ki67 (1:500; Abcam), and guinea pig anti-BrdU (1:200; Abcam); rabbit anti-calbindin (1:1000; CB 38, Swant); and rabbit anti-cleaved caspase-3 (1:250; Cell Signaling Technology). The sections analyzed for BrdU incorporation were pretreated with 2N HCl for 30 min, followed by 0.1 m boric acid for 15 min at room temperature. Following three washes with PBS for 10 min, sections were incubated with appropriate secondary antibodies diluted in carrier solution for 1 h. All secondary antibodies used were obtained from Jackson ImmunoResearch: Alexa Fluor-488-, Alexa Fluor-546-, or Alexa Fluor-647-conjugated donkey anti-rabbit, anti-goat, or anti-guinea pig antibodies (1:200).

### Dihydroethidium staining.

Pups received three subcutaneous injections of dihydroethidium (DHE; Thermo Fisher Scientific), diluted in saline at a dose of 3 mg/kg (injected volume, 10 µl/g body weight) before exposure to HO, and after 12 and 24 h of HO. P7 pups were transcardially perfused with 4% PFA; their brains were collected, postfixed, cryoprotected, and sectioned as described above.

### Bromodeoxyuridine administration.

To evaluate cellular uptake of bromodeoxyuridine (BrdU), both control and HO-treated mice were injected intraperitoneally with 50 mg/kg body weight BrdU for 2 h before being killed following a standard protocol (Schmitz et al., 2011). All mice were anesthetized with isoflurane and perfused with cold PBS followed by 4% PFA, and brain tissues were processed as described above.

### Confocal microscopy.

A Zeiss LSM 700 confocal laser-scanning microscopic system was used to analyze fluorescence-labeled cells (Zeiss Microscopy). Serial optical sections were acquired with a field depth of 2–5 µm, using a 20× or 40× objective. Four laser lines were used for the excitation of DAPI (400 nm excitation), Alexa Fluor-488 (488 nm excitation; 522/35 emission filter), Alexa Fluor-546 (555 nm excitation; 605/32 emission filter), and Alexa Fluor-647 (647 nm excitation; 680/32 emission filter). Data acquisition and processing were performed using LSM software. Cells were counted on bilateral hippocampi from three different coronal sections using the Cell Counter plugin of ImageJ/Fiji program (NIH).

### Administration of GSK3β inhibitor.

Pups received three subcutaneous injections of SB216763 (SB; Sigma-Aldrich) at P6, P7, and P8. The drug was dissolved in saline containing dimethylsulfoxide (25%; DMSO) and polyethylene glycol (25%; PEG). Mice were treated with either vehicle (DMSO/PEG in saline) or SB216763 (15 mg/kg). The injected volume was 10 µl/g body weight.

### Western blots.

Whole hippocampi were dissected out and homogenized in appropriate volumes of radioimmunoprecipitation assay lysis buffer consisting of the following (in mm): 50 Tris-HCl, pH 7.4, 150 NaCl, 2 EDTA, 50 NaF, 1 Na3VO4, 1% Triton X-100, 0.1% SDS, 0.5% Na-deoxycholate, and a Protease/Phosphatase Inhibitor Cocktail (Santa Cruz Biotechnology). Following centrifugation at 14,000 × *g* for 10 min, protein concentration was determined using the Bradford protein assay kit (BIO-RAD). Ten microliters of each protein sample containing 10 µg of total proteins was resolved by SDS-PAGE using 10% Bis-Tris precast gel (Thermo Fisher Scientific) and transferred to polyvinylidene fluoride membranes overnight at 4°C. Membranes were incubated with blocking buffer consisting of 4% nonfat milk in 1% Tween-20 in Tris-buffered saline (TBS-T) for 1 h, followed by overnight incubation at 4°C with one of the following primary antibodies diluted in 3% BSA TBS-T: rabbit anti-p(S473)AKT (1:500; catalog #4060, Cell Signaling Technology), rabbit anti-p(S9)GSK3β (1:1000; catalog #9323, Cell Signaling Technology), rabbit anti-p(Y216)GSK3β (1:1000; catalog #75745, Abcam), rabbit anti-cleaved caspase-3 (1:1000; catalog #9664, Cell Signaling Technology), rabbit anti-p(β-catenin) (1:1000; catalog #9561, Cell Signaling Technology), mouse anti-nitrotyrosine (1:1000; catalog #32–1900, Thermo Fisher Scientific), rabbit anti-NQO1 (1:1000; catalog #80588, Abcam), rabbit anti-HO-1 (1:1000; catalog #ab52947, Abcam), and mouse anti-β-actin (1:3000; catalog #MABT825, Millipore). After three washes with TBS-T, membranes were incubated with horseradish peroxide-conjugated secondary antibodies and protein bands were visualized using an enhanced chemiluminescence detection system (BIO-RAD) according to manufacturer instructions. Signal intensities of protein bands were quantified using ImageJ software and were normalized with actin as an internal control.

### Real-time quantitative PCR.

Total RNA was extracted from whole hippocampi using the RNeasy Mini Kit (Qiagen) according to manufacturer instructions. Real-time quantitative PCR (qPCR) was performed as previously described (Chew et al., 2013). Briefly, 200 ng of total RNA was used to synthesize cDNA in a 20 µl volume using the iScript cDNA Synthesis Kit (BIO-RAD). Five nanograms of each first-strand cDNA (in quadruplicates) was amplified in a 20 µl reaction mix containing SsoAdvanced Universal SYBR Green Supermix (BIO-RAD) and 1 mm forward and reverse primers using the CFX96 Real-Time System (BIO-RAD). The cycling parameters used were as follows: 95°C for 10 min, followed by 40 cycles of 95°C for 45 s, 55°C for 45 s, and 72°C for 45 s. Changes in mRNA expression were calculated using the ΔΔCt method with actin serving as an internal control. The following primers used were obtained from Integrated DNA Technologies: NRF2 forward, 5′-GGAGGCAGCCATGACTGA-3′; NRF2 reverse, 5′-CTGCTTGTTTTCGGTATTAAGACT-3′; actin forward, 5′-ATGCTCCCCGGGCTGTAT-3′; and actin reverse, 5′-CATAGGAGTCCTTCTGACCCATTC-3′.

### Microarray.

RNA was isolated from the whole hippocampus of mice using the RNeasy Mini Kit (Qiagen) according to the manufacturer instructions and quantified using a spectrophotometer (model ND-1000, NanoDrop Technologies). RNA quality was analyzed by determining the RNA Integrity Number (RIN) using a bioanalyzer (model 2100, Agilent Technologies). High-quality RNA (with RIN of at least 6) was used to perform an expression profile of mRNA using the Gene Expression BeadChip Array (Illumina) according to the manufacturer guidelines. In brief, the cRNA library was first generated from mRNA and amplified using an RNA amplification kit (Illumina TotalPrep-96, Ambion). Biotin-linked nucleotides were hybridized to the HumanHT-12-v4-BeadChip (Illumina) for 16 h, sequentially followed by washing, blocking, and staining with Streptavidine-Cy3 following the Expression Direct Hybridization protocol (Illumina). Arrays were scanned using the HiScanSQ System, and images were analyzed by GenomeStudio Gene Expression Module (Illumina). Data were analyzed using one-way ANOVA at *p* < 0.05. Heatmap visualization of the expression data were performed using the heatmap.2 function from the gplots package (Warnes et al., 2005).

### Single-cell RNA-sequencing.

Hippocampi were dissociated with the Papain Dissociation System (Worthington) and captured with the Fluidigm C1 Single-Cell mRNA Seq HT system together with the medium size chip (which is optimal for cells at 10–17 µm) according to the manufacturer instructions. Immediately before cell capture, viability was assessed by trypan blue staining. The cell viability was close to 90%. After the capture, the C1 chip was examined visually, and the number of cells and cell viability at each capture site were recorded manually. Cells captured by C1 were subsequently processed through lysis, reverse transcription, and PCR amplification to generate single-cell full-length cDNA using the Smarter Ultra Low Input RNA kit for Fluidigm (Clontech). The cDNAs from all capture sites were harvested 18 h later and subjected to downstream processing for sequencing-ready library preparation, followed by deep sequencing using the HiSeq 2500 sequencing system (Illumina), followed by demultiplexing and trimming of the sequencing reads. The sequencing reads were mapped to the mouse reference genome (assembly GRCh38.78) using TopHat version 2.1.0 (Trapnell et al., 2009). Read counts per gene were obtained with the Cufflinks version 2.2.1 suite (Trapnell et al., 2010). All RNA-sequencing (RNA-seq) data have been uploaded to the Sequence Read Archive (National Center for Biotechnology Information; accession code PRJNA816085).

### Bioinformatic analysis.

The read counts data from Cufflinks were loaded into a Seurat (Stuart et al., 2019) object with a metadata mapping file. The data were then processed in a standard Seurat (version 3.2.2) analysis pipeline. Briefly, the data were log normalized before highly variable genes (hvgs) were empirically detected using the FindVariableFeatures function-based mean expression level and variance of the genes. The top 2000 genes with the highest variance at an expression level >0.23 were regarded as hvgs. Then, normalized read counts were scaled, and principal component analysis was performed with the hvgs. The first 10 principal components were used to perform a two-dimensional Uniform Manifold Approximation and Projection (UMAP) dimension reduction. Clustering was conducted using the Louvain–Jaccard method on the UMAP coordinates. The clusters were manually annotated as cell types by rereferring to cluster markers. Differentially expressed genes in each cell type were identified with the FindAllMarkers function. The annotated clusters were further inspected for differential gene expression between control and hyperoxia cells using the function FindMarkers. The heatmap was created using the function heatmap.3. Violin plots were made with ggplot2 (Wickham, 2016).

### Water T-maze.

The T-maze apparatus consisted of 35-cm-long and 10-cm-wide arms made from translucent PVC (polyvinyl chloride) material. The maze was filled with water up to a depth of 21 cm, with temperature maintained between 21 and 23°C. The platform was a 5 × 5 cm rectangular Plexiglas box designed to fit into each arm of the maze such that a 1 cm water level is maintained above it. The study consisted of the following three phases: pretraining preference determination, training/memory acquisition, and test/reverse-learning phases. During the pretraining preference determination phase, mice were placed at the foot of the maze and allowed to swim until they reached one T-maze arm. The direction of the arm each mouse turned to (left or right) was noted. The trial was repeated eight times, and the preferred direction was determined based on the most frequent arm in which the mouse turned. During the first day of training, a hidden platform was placed in one arm of the maze, and mice were placed at the foot of the maze and allowed to swim for 60 s to locate the platform. The location of the platform was opposite the direction of the preferred orientation of turn of each mouse. Mice that failed to locate the platform were gently guided to the platform. All mice were allowed to remain on the platform for 5 s before being returned to the home cage. The training session was repeated eight times. On subsequent post-training days, mice were assessed for their ability to locate the platform on pass criteria, which include an ability (1) to correctly locate the hidden platform and (2) to remain on the platform for 5 s. Mice that met the pass criteria on at least seven of eight total trials per day were considered to have passed the test. Mice that met the pass criteria for at least 3 consecutive days were then tested for reversal learning. During reverse learning, the hidden platform was placed in the other arm of the maze opposite the arm used during the test phase. All tests were performed by an observer blinded to the treatment.

### Novel object recognition test.

The test field was composed of a rectangular-shaped box (12 × 6 cm). Before testing, mice were subjected to short habituation in which test rodents were placed in the Plexiglas box for 15 min before being returned to their home cage. On the following day, mice were placed in the test field with two identical objects and allowed to explore the field for a maximum of 10 min. The time that a mouse spent exploring each identical object was noted. After familiarization, mice were returned to home cages for 6 or 24 h before the test phase. During testing, one of the identical objects was replaced with a novel object of distinctively different shape, texture, and size, and the times that mice spent exploring the novel and known objects were noted. The degree of discrimination was determined by the length of time a mouse spent with the novel object. Regardless of the total time spent, mice were deemed to have completed the familiarization training if the time spent exploring both identical objects reached 20 s.

### Ca^2+^ imaging and analysis.

Two-photon Ca^2+^ imaging was performed with an imaging system (FluoView FVMPE-RS Multiphoton Microscope, Olympus) using FluoView software and a Ti:Sapphire laser source emitting 140 fs pulses at an 80 MHz repetition rate with a wavelength adjustable for 690–1040 nm (MaiTai DeepSee pulsed, infrared laser). Full-field view images were acquired with XY raster scanning using the 20× (0.95 numerical aperture) water-immersion objective. Changes in fluorescence (Δ*F*) were quantified using ImageJ (NIH) software and were expressed as a percentage of baseline (%Δ*F*/*F*). Time-lapse images of GAD2CreER^T2^/GCAMP5TdTomato interneuron Ca^2+^ signaling were recorded at a frame rate of 1 Hz. Regions of interest (ROIs) were selected based on the appearance of GCaMP5G Ca^2+^ transients in the time-lapse images. To trigger Ca^2+^ transients, the designer receptors exclusively activated by designer drugs (DREADDs) agonist clozapine-*N*-oxide (CNO; 100 µm) was dissolved in artificial CSF (ACSF) and delivered locally by a pressure pulse (10 psi; 100–500 ms) using a Picospritzer III (Parker Instrumentation).

### Electrophysiology.

Mice were anesthetized with isoflurane and decapitated, and the brains were dissected into ice-cold cutting ACSF bubbled with 95% O_2_ and 5% CO_2_ (Carbogen). The ACSF was composed of the following (in mm): 2 MgSO_4_, 2 MgCl_2_, 1 CaCl_2_, 1.25 NaH_2_PO_4_, 2 KCl, 234 sucrose, 25 NaHCO_3_, and 10 glucose. Adult (P60) mice were perfused with aerated ice-cold ACSF before decapitation. Serial coronal slices (300 µm thick) containing hippocampus were generated using a vibratome (model VT 1200 S, Leica). Slices were allowed to recover for at least 30 min in a holding chamber with continuously aerated incubation ACSF composed of the following (in mm): 125 NaCl, 2 KCl, 2 MgSO_4_, 1 CaCl_2_, 1.25 NaH_2_PO_4_, 20 sucrose, 25 NaHCO_3_, and 10 glucose. Whole-cell patch-clamp recordings were performed on pyramidal cells and interneurons in the CA1 of hippocampus in coronal slices in a submersion recording chamber perfused with recording ACSF of the following composition (in mm): 125 NaCl, 2 KCl, 1 MgSO_4_, 2 CaCl_2_, 1.25 NaH_2_PO_4_, 25 NaHCO_3_, and 10 glucose. All recordings were performed with a pulled borosilicate glass pipette (4–7 µm) filled with internal solutions, the choice of which reflected the current being analyzed. IPSCs were recorded in voltage-clamp mode at a holding potential of −70 mV with an internal solution composed of the following (in mm): 135 Cs-gluconate, 10 MgCl2, 0.1 CaCl2, 1 EGTA, 10 HEPES, 2 Na-ATP, and 0.2 Na3 GTP, at pH 7.3 (280–290 mOsm). For recordings of firing properties (performed in a current-clamp mode) and EPSCs (performed in a voltage-clamp mode), the internal solution was composed of the following (in mm): 130 K-gluconate, 2 MgCl2, 10 EGTA, 10 HEPES, 2 Na-ATP, and 0.2 Na_3_ GTP, at pH 7.3 (280–290 mOsm). All internal solutions had a pH of 7.3 and an osmolarity of 280–290 mOsm. For recordings of viral-transfected hippocampal interneurons *in vitro*, we used Gad2Cre mice transduced with either a DREADD or channelrhodopsin (ChR)-expressed adeno-associated virus 2 (AAV2; see below), and all recordings were performed in current-clamp modes. All recorded currents were amplified using a Muticlamp 700B amplifier, filtered at 2 kHz, digitized with a Digidata 1322A digitizer (Axon, Molecular Devices), and analyzed offline using Clampfit 10.2 (Axon, Molecular Devices). The immunofluorescent detection of staining p(Y216)-GSK3β was conducted in similar P8 hippocampus slices acutely exposed *ex vivo* to HO for 20 min (pO_2_ > 500 mmHg) before fixation and resectioning for histologic analysis as previously described (Papazoglou et al., 2015).

### Stereotaxic injection of double-floxed inverted open reading frame recombinant AAV.

Gad2Cre mice of both sexes were anesthetized with a 10:1 mixture of ketamine/xylazine in saline such that each mouse received 100 mg/kg ketamine and 10 mg/kg xylazine, respectively. After anesthesia was confirmed through tail pinch, mice heads were shaved and placed onto a stereotaxic frame (Stoelting). An anterior–posterior skin incision was made, and a craniotomy hole was drilled bilaterally through the skull at the injection site. An injection of 1.0 µl of pAAV-hSyn-DIO-hM3D(Gq)-mCherry (AAV2 DREADD) or pAAV-EF1a-DIO-hChR2(H134R)-mCherry (channelrhodopsin) virus was administered bilaterally into the CA1 using a Hamilton microsyringe using the following coordinates: 2 mm posterior to bregma, 1.5 mm lateral to the midline, and 2 mm deep from the dura. After injection, the Hamilton syringe was allowed to remain in place for 5 min and was withdrawn slowly to allow for diffusion of the virus within the CA1 region while preventing nonspecific labeling of cortical interneurons. For experiments involving *in vivo* optogenetic stimulation of Gad2 interneurons, optogenetic fibers were also simultaneously planted in the CA1 during the viral injection. Following viral injection, craniotomy holes and skin scalp were sealed with styptic power [Kwik stop, ARP (in mice with implanted optogenetic fiber only)] and Vetbond (3M), respectively. Mice were allowed to recover in their home cage maintained at 37°C until recovery from anesthesia. Throughout the period of recovery from surgery, mice were provided with buprenorphine-treated chow for pain control.

### *In vivo* optogenetic and chemogenic stimulation of Gad2 hippocampal interneurons.

*In vivo* optostimulation of Gad2Cre^+^ cells was performed using a 40 kHz OmniPlex D (version 1.11; Plexon) neural data acquisition system and preamplified using a MiniDigi preamplifier (16 channels; Plexon). The experimental protocol used was modified from a previously described procedure (Sathyanesan et al., 2018). In summary, a 25 Hz pulse train was bilaterally applied to hippocampal CA1, each with a duration of 30 s. A total of 25 pulses was delivered per treatment at intervals of 30 s. The initial phase of the optogenetic stimulation was performed with mice placed in an empty rectangular-shaped Plexiglas box (12 × 6 cm) used for the novel object recognition test (NORT). During the last 10 min of stimulation, mice were transferred into a similar apparatus with identical objects to allow for the performance of the familiarization phase of the NORT. Control mice were not subjected to optogenetic stimulation. Chemogenetic stimulation of DREADD virus-transfected Gad2Cre mice was performed through intraperitoneal injection of CNO at a dose of 50 mg/kg body weight 45 min before the familiarization test of the NORT. Control mice were injected with saline. Learning behavior was performed 6 h postoptogenetic or chemogenic stimulation as described above.

### Statistical analysis.

Appropriate parametric tests were applied to analyze differences between treatment groups. Data were expressed as the mean ± SEM, and differences were analyzed using an independent *t* test or one-way ANOVA followed by Bonferroni's or Tukey's *post hoc* tests. Cumulative probability distributions were analyzed with the Kolmogorov–Smirnov test.

## Results

### Perinatal hyperoxia causes oxidative stress in the developing hippocampus

**Figure 1.**
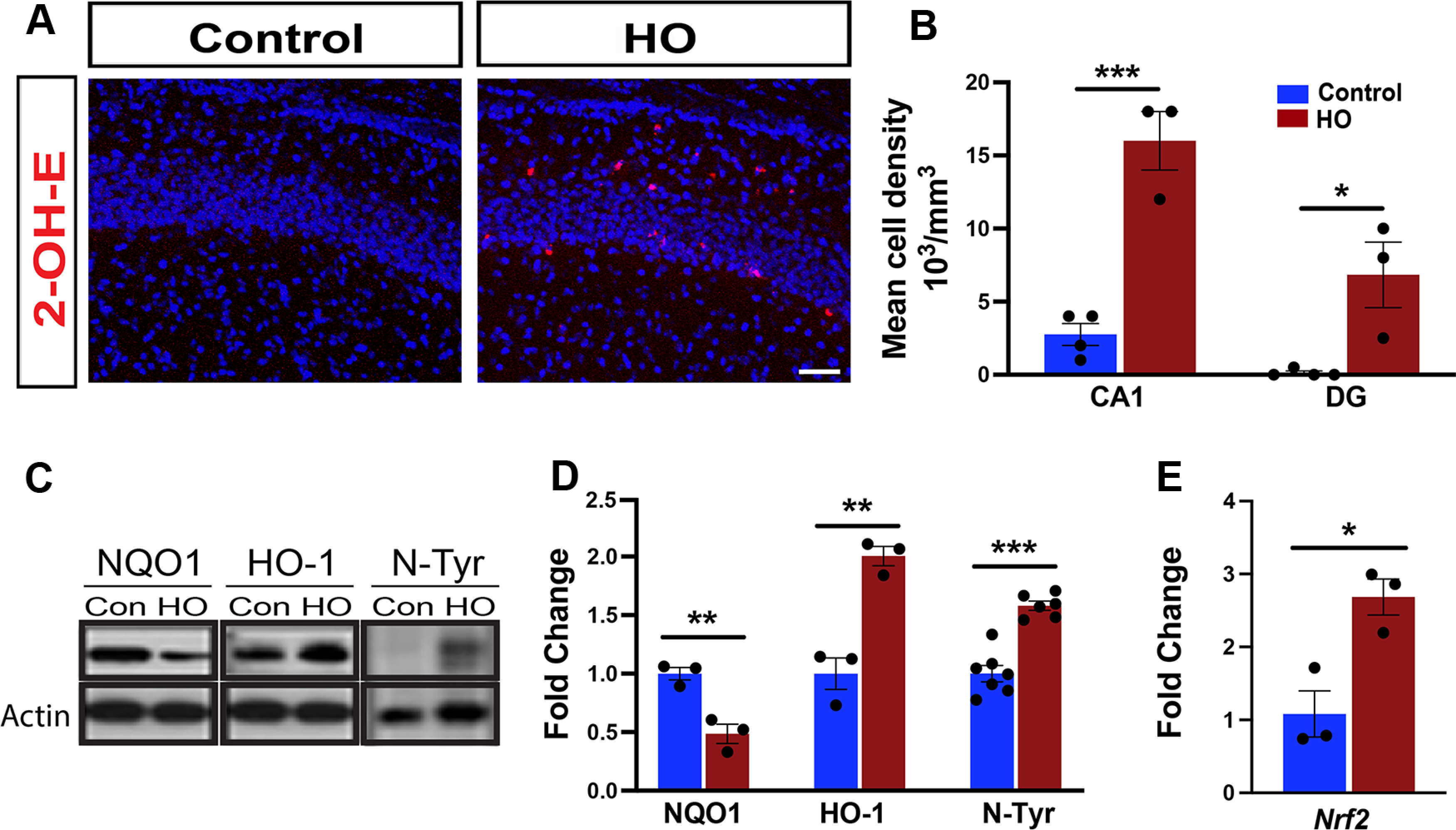
Perinatal HO increases oxidative stress in the hippocampus. ***A***, Representative images showing 2-OH-E^+^ staining (red), the oxidative product of DHE, and DAPI (blue), indicating the production of superoxide anion in the P7 mouse CA1. ***B***, Quantification of 2-OH-E^+^-expressing cells in the CA1 and DG. ***C***, Western blots of NQO1, HO-1, and *N*-Tyr from whole P8 hippocampal samples. ***D***, Quantification of protein levels of NQO1, HO-1, and *N*-Tyr from Western blots. ***E***, qPCR quantification of *Nrf2* mRNA expression in P8 hippocampus. Con, Control. Control versus HO, Student's unpaired *t* test, *p < 0.05; **p < 0.01; ***p < 0.005. Scale bar, 150 µm.

We hypothesized that increased oxygen tension would overwhelm neonatal antioxidant defenses and increase oxidative stress in the developing brain. Using our neonatal rodent injury model of 80% oxygen between P6 and P8 (Schmitz et al., 2011), we evaluated indicators of oxidative stress in the hippocampus. DHE is a cell-permeable dye that reacts with superoxide anion (O_2_^•−^) in tissues (Bindokas et al., 1996) to form a red fluorescent ethidium (Rothe and Valet, 1990; Carter et al., 1994) or 2-hydroxyethidium (2-OH-E^+^; Zhao et al., 2005), which intercalates into DNA and can be detected by microscopy. The number of 2-OH-E^+^ cells significantly increased following 24 h of exposure to HO, indicating an increased level of superoxide radicals (Fig. 1*A*,*B*). This increase is detected in the CA1 and DG (Fig. 1*B*). NAD(P)H dehydrogenase (Quinone) 1 (NQO1) provides antioxidant defense, and its reduction increases the risk of neuronal damage (Luo et al., 2019). Heme oxygenase (HO-1) regulates heme degradation, a process upregulated by cellular stress (Choi and Alam, 1996). Nitrotyrosine (N-Tyr) is generated from peroxynitrite, a product of the reaction between superoxide anion and nitric oxide. Its levels correlate with the accumulation of ROS (Beckman et al., 1996; Crow and Beckman, 1996). At P8, hippocampal levels of all three oxidative stress markers—NQO1, HO-1, and *N*-Tyr—respond significantly to high oxygen levels, indicating loss of antioxidant activity (NQO1), increased stress (HO-1), and ROS activity (N-Tyr; Fig. 1*C*,*D*). Furthermore, quantitative real-time PCR assays revealed that gene expression of the nuclear factor erythroid 2-related factor 2 (*Nrf2*), a master regulator of oxidative stress-responsive genes (Zhang et al., 2015), is significantly upregulated in hippocampal tissue at P8 (Fig. 1*E*). These results indicate that HO exposure during early postnatal development causes oxidative stress in the hippocampus.

### High oxygen disrupts hippocampal neuron development and causes cell death

**Figure 2.**
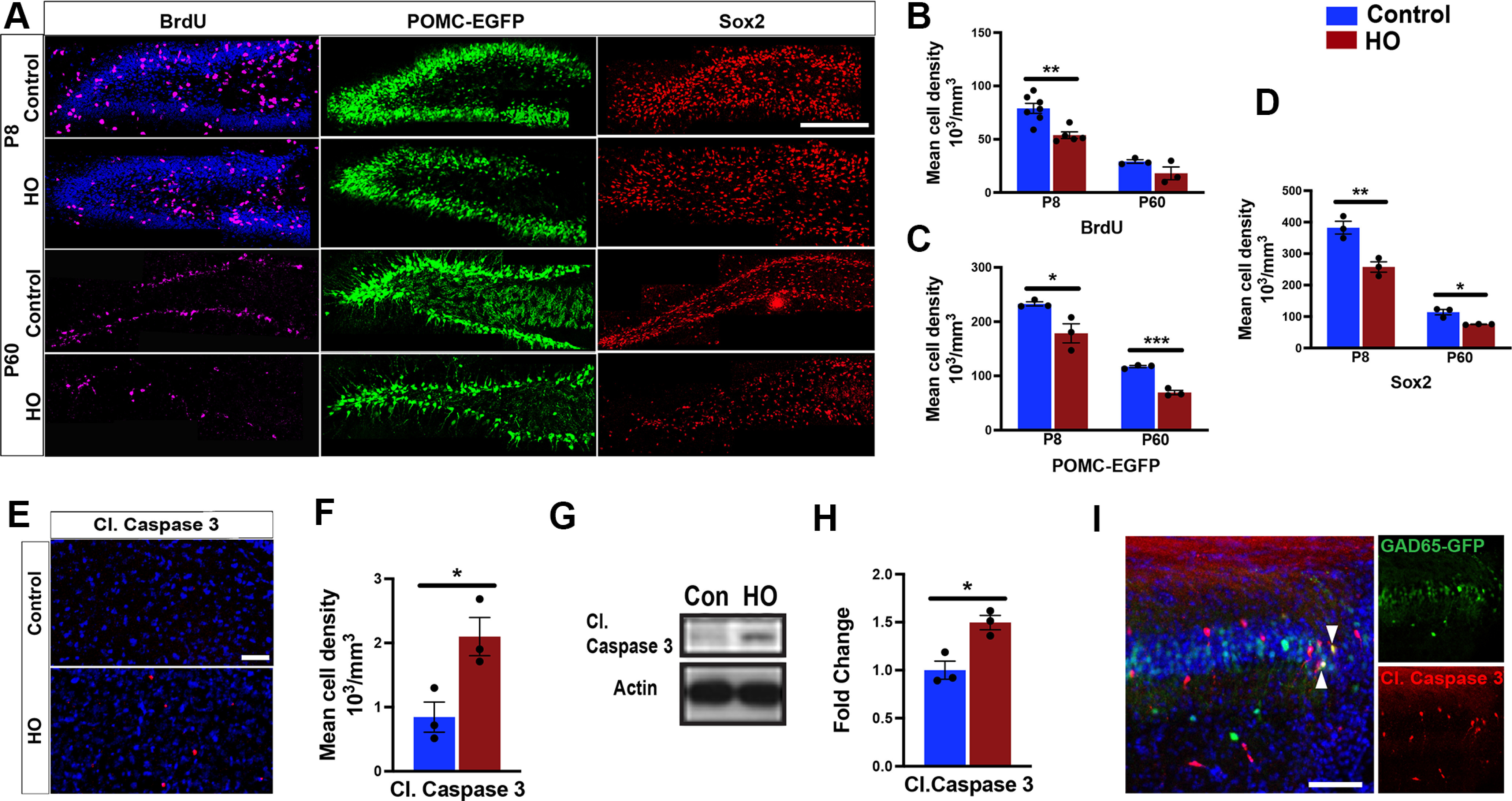
Perinatal oxidative stress disrupts hippocampal development. ***A***, Representative images of BrdU^+^ (magenta), POMC-EGFP^+^ (POMC; green), and Sox2+ (red) immunostaining in the DG at P8 and P60. ***B–D***, Mean densities of BrdU^+^ (***B***), POMC-EGFP^+^ (***C***), and Sox2^+^ (***D***) cells in the hippocampus. ***E***, Representative images of cleaved caspase-3-expressing cells (red) in P8 hippocampus. ***F***, Mean densities of cleaved caspase-3-expressing cells in P8 hippocampus. ***G***, Western blot analysis of cleaved caspase-3 in P8 hippocampus. ***H***, Quantification of protein expression levels of cleaved caspase-3 from Western blotting in ***G***. ***I***, Representative image of GAD65-GFP (green) and cleaved caspase-3-coexpressing cells (red) in P7 CA1 during HO. Con, Control. Student's unpaired *t* test, control versus hyperoxia; *p < 0.05; **p < 0.01; ***p < 0.005. Scale bars: ***A***, 150 µm; ***E***, 100 µm; ***I***, 100 µm.

Based on the evidence of HO-induced oxidative stress in the hippocampus, we hypothesized that cellular changes involve alterations in cell proliferation and viability, which are a common substrate in neurodevelopmental disorders (Li et al., 2019). First, we analyzed the neurogenic niche of the DG by administering BrdU 2 h before tissue collection. Interestingly, HO significantly reduced the number of BrdU^+^ cells at P8 (Fig. 2*A*,*B*), which was accompanied by a decline in POMC-EGFP^+^ newly born granule cells and Sox2-expressing progenitor cells at both P8 and P60 (Fig. 2*C*,*D*). In addition to impaired neurogenesis, apoptosis, as indicated by increased numbers of cleaved Caspase-3-expressing cells and hippocampal protein level, was evident at P8, likely contributing to a reduction in hippocampal neurons (Fig. 2*E–H*). Indeed, multiple GAD65-expressing interneurons coexpressed cleaved caspase-3 in the CA1 of GAD65-GFP mice after 24 h of HO (Fig. 2*I*).

**Figure 3.**
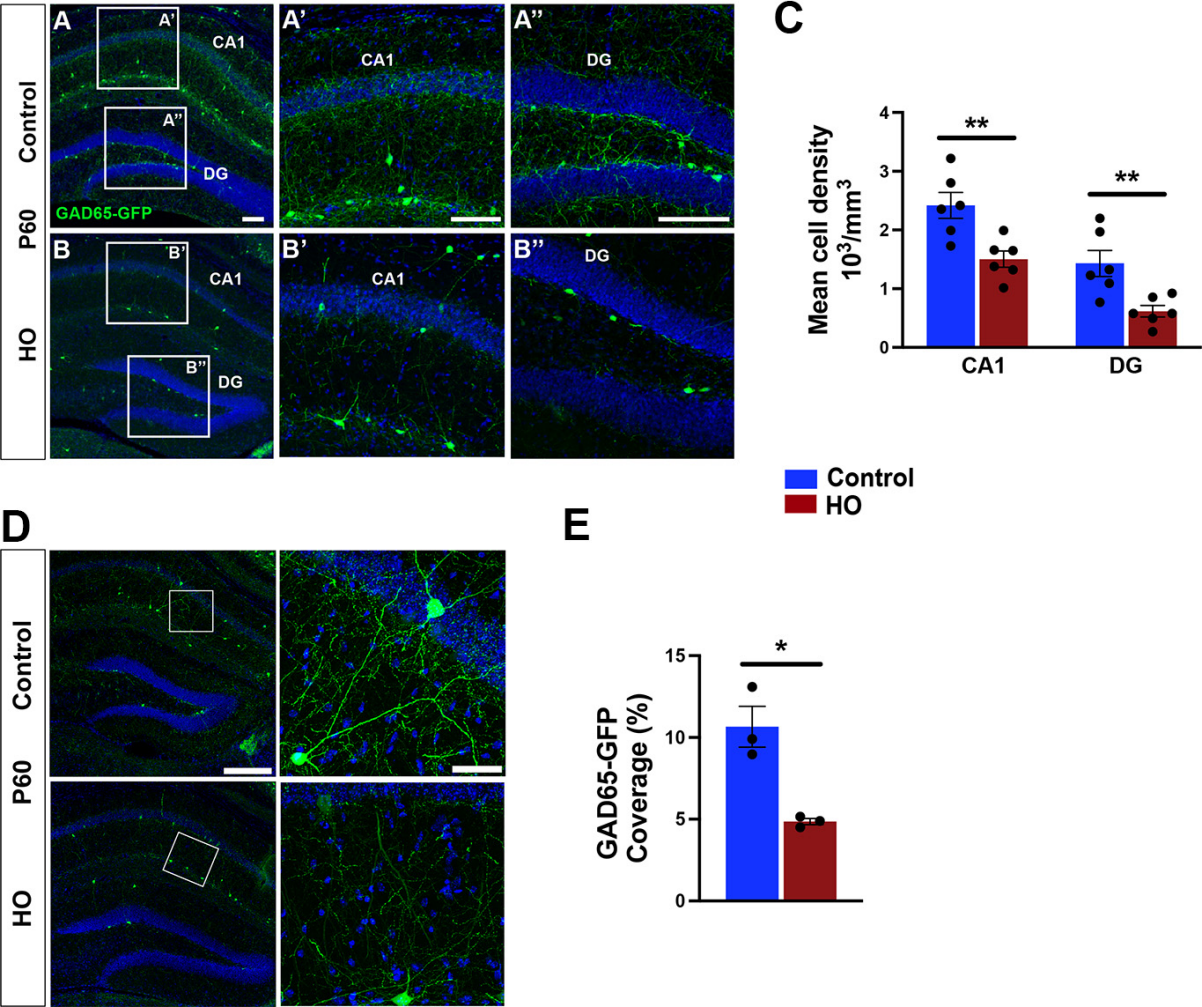
Perinatal oxidative stress causes long-term alteration in interneuron density and arborization. ***A–B″***, Representative images of GAD65-GFP neurons (green) in P60 CA1 and DG in control mice (***A***) and HO-exposed mice (***B***); boxes indicate magnified areas (***A′***, ***A″***, ***B′***, ***B″***). ***C***, Quantification of GAD65-GFP neuron density in P60 CA1 and DG. ***D***, Dendritic arborization of GAD65-GFP neurons in the CA1 of control and HO-exposed mice. Box indicates magnified area. ***E***, Quantification of the area covered by GAD65-GFP neurons in the hippocampus at P60. Student's unpaired *t* test, control versus hyperoxia: *p < 0.05; **p < 0.01. Scale bars: ***A–B″***, 100 µm; ***D***, left, 300 µm; ***D***, right, 50 µm.

Because inhibitory neurons are commonly affected in neurodevelopmental disorders (Benes et al., 1998; Konradi et al., 2011; Schobel et al., 2013; Caletti et al., 2015; Reichel et al., 2015), we next asked whether HO produced long-term changes in the GAD65-expressing interneuron density of GAD65-GFP mice. At P60, the number of GAD65-GFP^+^ interneurons was significantly decreased by HO in the CA1 and DG (Fig. 3*A–C*). In addition, hippocampal GAD65 neuron dendritic arborization was altered by HO (Fig. 3*D*), resulting in reduced dendritic coverage at P60 (Fig. 3*E*).

**Figure 4.**
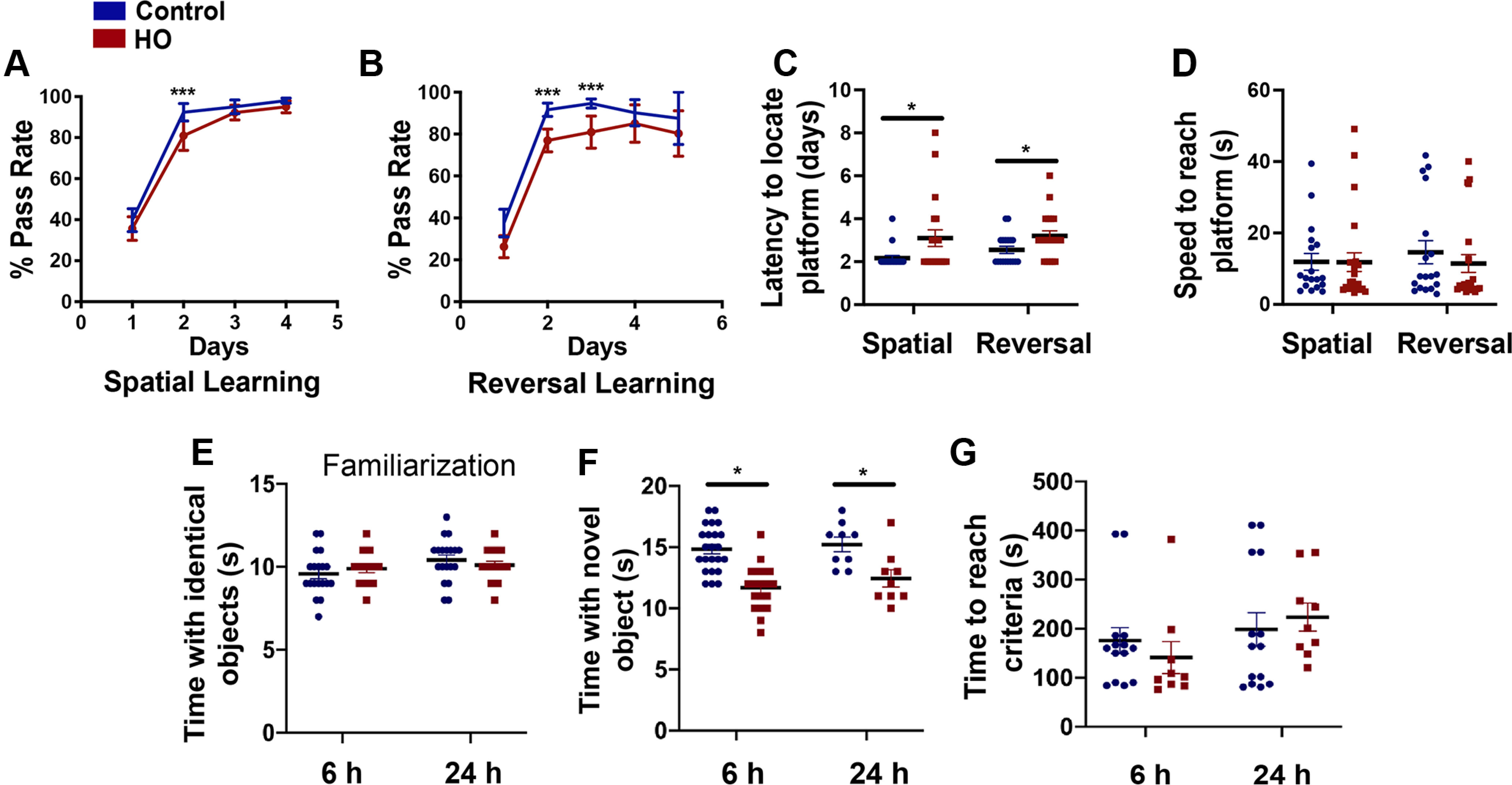
HO causes hippocampus-dependent learning deficits. ***A***, ***B***, In the water T-maze test, quantification of the percentage pass rate to locate the platform during the acquisition (***A***) and reversal learning (***B***) phases in control (blue) and HO (red) mice. ***C***, Quantification of the number of days to reach criteria during the spatial learning phase (acquisition) and reversal learning phase. ***D***, The kinetic behavior (speed to reach the platform) measured in control and HO-exposed mice. ***E***, In the novel object recognition test, quantification of the time spent exploring identical objects at 6 and 24 h during the familiarization phase. ***F***, Quantification of the time spent exploring novel objects at 6 and 24 h following the familiarization phase. ***G***, Quantification of the time taken to reach criteria (completed exploration tasks of 20 s) in control and HO mice. ***A***, ***B***, Two-way ANOVA with Tukey's *post hoc* test, ***p < 0.005. ***C–G***, Student's unpaired *t* test, control versus HO, *p < 0.05.

To understand the functional consequences of these neuronal changes, behavioral assays to determine cognitive ability were performed at P60. The water T-maze test showed that spatial memory acquisition and reversal learning were significantly impaired following HO (Fig. 4*A*,*B*). Also, injured mice took more days to find the platform in both learning paradigms, despite there being no differences in the speed to reach the platform (Fig. 4*C*,*D*). In the NORT, the familiarization phase and time to reach criteria were indistinguishable between groups, but the time spent with the novel object was reduced in the HO group, indicating that injured mice have impaired recognition memory (Fig. 4*E–G*). These assays support the interpretation that hippocampus-dependent behavioral processes are compromised at P60 following early exposure to oxidative stress.

### Hippocampal interneuron stimulation reverses high oxygen-induced reduction in spike frequency and learning deficits

**Figure 5.**
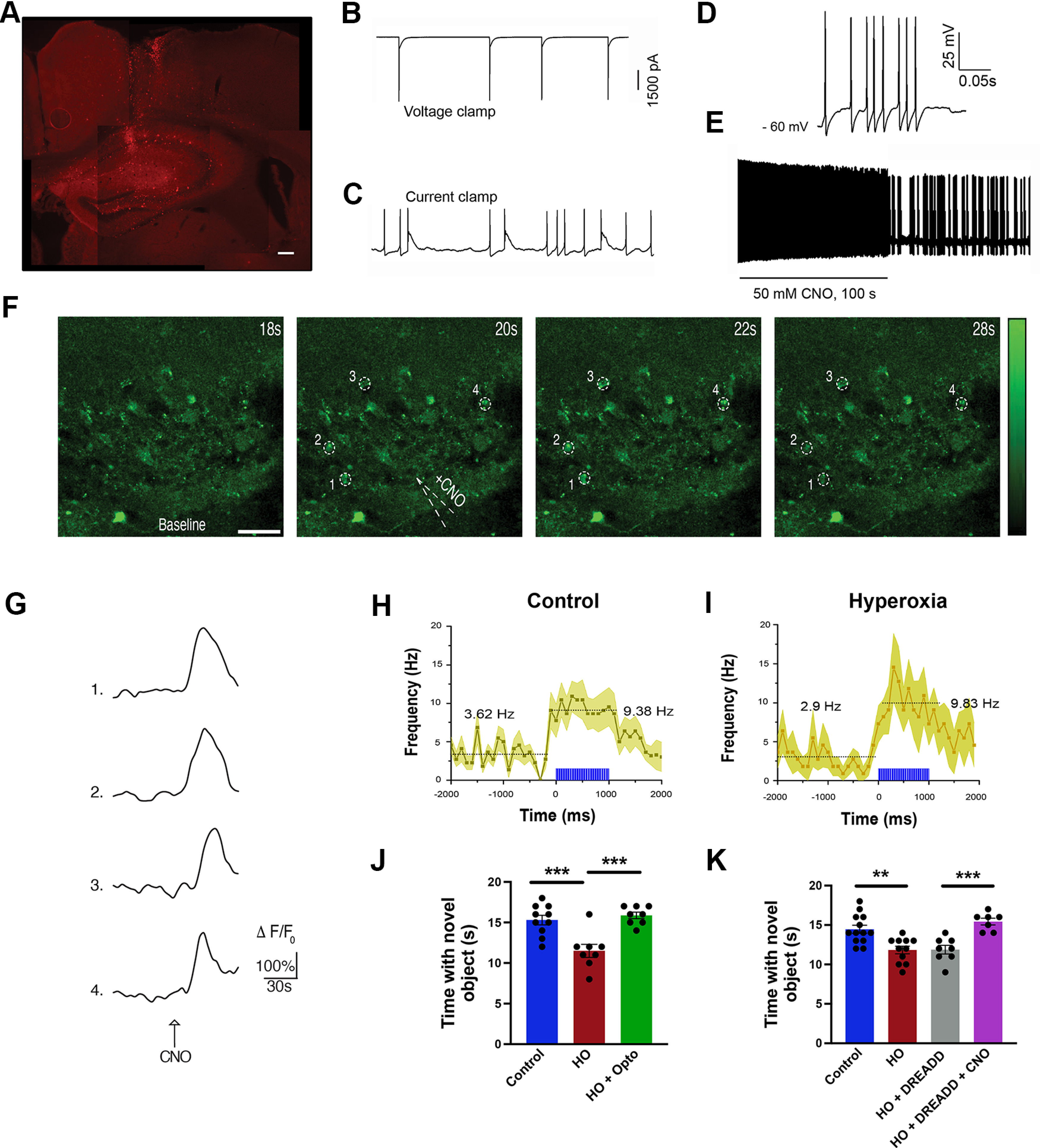
Optogenetic and chemogenetic stimulation of hippocampal interneurons rescues HO-induced learning deficits. ***A***, Representative image of coronal slices of P40 Gad2Cre mice injected with pAAV-EF1a-DIO-hChR2(H134R)-mCherry virus shows the route of virus delivery in the CA1 layer with ChR2-mCherry (red) expression throughout CA1. Scale bar, 50 µm. ***B***, ***C***, Action potential recording of Gad2Cre^+^ cells from tissue slice similar to that in ***A*** in voltage-clamp mode (***B***) and current-clamp mode (***C***) without light stimulation. ***D***, *In vitro* whole-cell patch-clamp recording of Gad2Cre^+^ cells in tissue slice from Gad2Cre mouse transduced with pAAV-hSyn-DIO-hM3D(Gq)-mCherry DREADD virus at P35 without CNO stimulation. ***E***, *In vitro* whole-cell patch-clamp recording of Gad2Cre^+^ cells that were transduced with pAAV-hSyn-DIO-hM3Dq(Gq)mCherry DREADD virus at P35 and stimulated with CNO at P45. ***F***, Time series of intracellular calcium increases induced by focal application of CNO (100 μm) from a glass pipette (white dotted line) in an acute brain slice prepared from Gad2Cre/GCaMP5G-tdTM mice transduced with pAAV-hSyn-DIO-hM3Dq(Gq)mCherry DREADD virus. The pseudocolor scale displays relative changes in GCaMP5G emission. Scale bar, 60 μm. ***G***, Representative individual traces of GCaMP5G fluorescence changes in (Δ*F*/*F*_0_) in response to CNO administration. White dotted circles in ***F*** correspond to the regions of interest in the GAD2Cre/GCaMP5G-tdTM^+^ cells. ***H***, ***I***, *In vivo* recording of P40 Gad2Cre^+^ cells in the hippocampus of mice transduced with pAAV-EF1a-DIO-hChR2(H134R)-mCherry, showing reduced prestimulation basal activity of cells in mice exposed to HO compared with control. Optogenetic stimulation (blue bars), raised activities in both groups to similar levels. ***J***, Optogenetic stimulation of Gad2Cre cells in the hippocampus rescued the deficit in object recognition in mice exposed to HO. ***K***, Chemogenetic stimulation of Gad2Cre cells in the P45 hippocampus with CNO rescued the deficit in object recognition in mice exposed to HO. One-way ANOVA with Tukey's *post hoc* test: **p < 0.005, ***p < 0.001.

Because of the deficits in hippocampal development and cognition, we reasoned that dysmaturation of GAD65^+^ interneurons impaired inhibitory neurotransmission. To determine whether hippocampal injury led to changes in interneuron function, we used optogenetic and chemogenetic approaches to stimulate interneurons. We expressed pAAV-EF1a-double floxed-hChR2(H134R)-mCherry virus that carries a light-activated cation channel (ChR) in the hippocampi of Gad2Cre mice, or DREADDs (pAAV-hSyn-DIO-hM3D(Gq)-mCherry) in the hippocampi of Gad2Cre/GCaMP5G-tdTM mice. We verified the virus-mediated expression of ChR in P40 Gad2Cre hippocampal slices (Fig. 5*A*) and recorded action potentials (Fig. 5*B*,*C*). Stimulation of brain slices from these animals with light pulse produced action potentials (Fig. 5*D*). In slices transduced with DREADD-Gq, CNO exposure also produced action potentials as well as an increase in calcium, as measured by GCaMP5G emission (Fig. 5*E–G*). Interestingly, we found that, although HO lowered spiking frequency in the P40 CA1 *in vivo* (Fig. 5*H*), optogenetic stimulation of Gad2Cre cells restored spiking frequency (Fig. 5*I*), indicating the capacity for recovery of electrophysiological function using postinjury stimulation of Gad2-expressing interneurons. Importantly, optogenetic stimulation of Gad2Cre interneurons concomitant with the familiarization phase of the NORT assay recovered the HO-induced deficit in hippocampus-dependent recognition memory (Fig. 5*J*). We confirmed the effect of postinjury interneuron stimulation by specifically activating Gad2Cre cells in mice transduced with DREADD-Gq. Similar to optogenetic stimulation, chemogenetic activation by CNO administration 45 min before the familiarization phase of the NORT assay significantly improved the HO-induced cognitive deficit in recognition memory (Fig. 5*K*).

### High oxygen alters signaling pathways in the developing hippocampus

**Figure 6.**
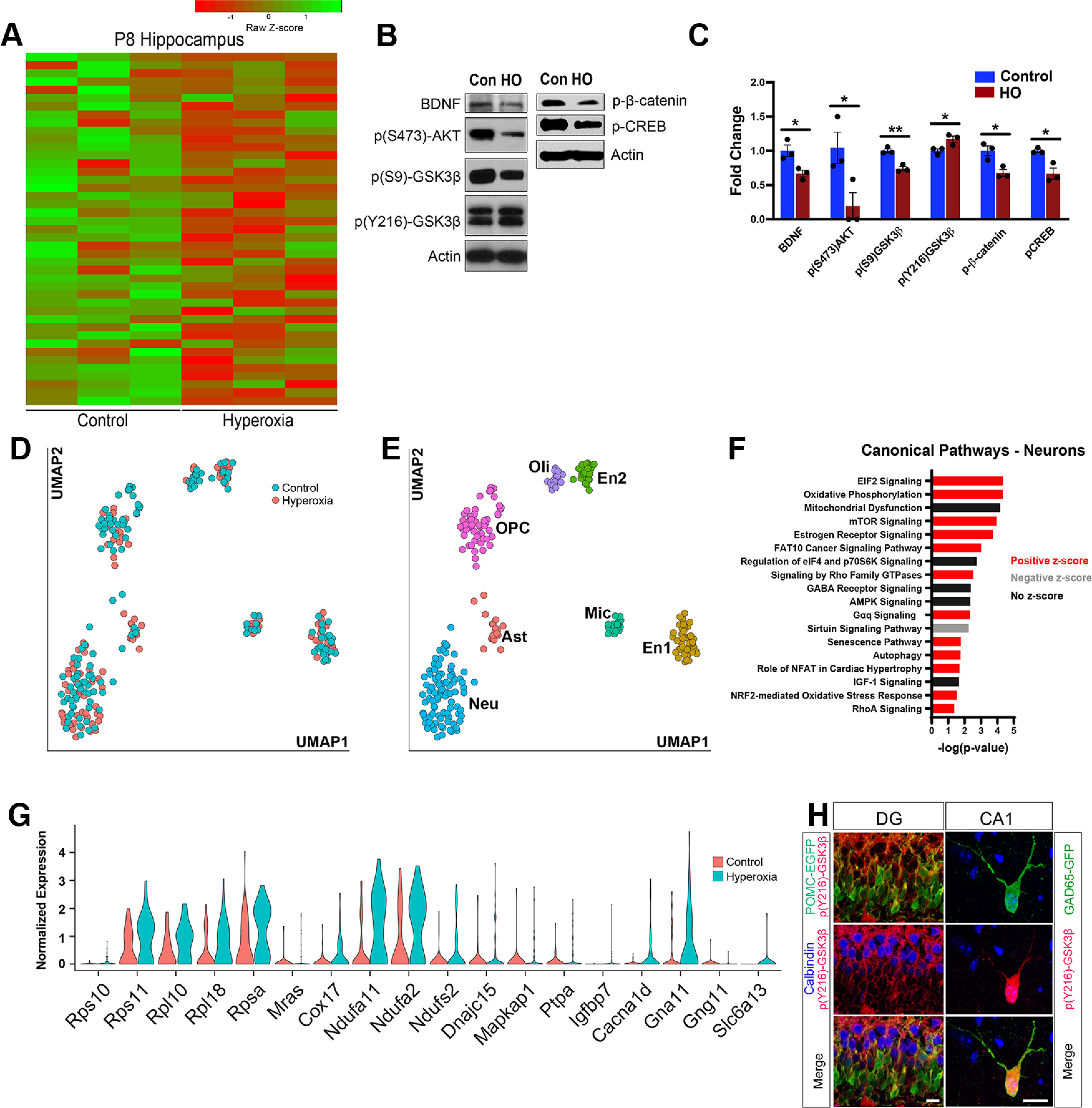
Gene expression analysis reveals HO-induced oxidative stress and altered neuronal development associated with dysregulation of Wnt and Akt signaling. ***A***, GeneChip microarray analysis of P8 hippocampus tissue showing differentially expressed genes as a heatmap. One-way ANOVA. Differential expression threshold: *p* < 0.05 and 1.3-fold change. ***B***, Western blot analysis of P8 hippocampal lysates showing the regulation of proteins associated with neurotrophin, Akt, and Wnt signaling. Con, Control. ***C***, Quantification of Western blots showing relative abundance of proteins. Student's *t* test. *p < 0.05; **p < 0.01. ***D***, Scatterplot of single-cell RNA-seq data in UMAP dimensions. Colors represent experimental conditions. ***E***, Scatterplot of single-cell RNA-seq data in UMAP dimensions. Colors represent cell types. ***F***, Top, Affected canonical pathways predicted based on differentially expressed genes in the Neuron cluster and ranked based on -log(*p*-value). ***G***, Violin plots of selected differentially expressed genes between conditions in the Neu cluster. ***H***, Dual-label immunohistochemistry of P8 hippocampus slices acutely exposed *ex vivo* to HO for 20 min (pO_2_ > 500 mmHg) before fixation and resectioning for histologic analysis. Images of DG (left column) and CA1 (right column) show colocalization of p(Y216)- GSK3β (red) with GAD65-GFP (green) or POMC-EGFP (green). CB cells of DG are in blue. Scale bars, 15 µm.

Since we found that HO-induced markers of oxidative stress in the hippocampus, we pursued signaling pathways whose dysregulation would lead to changes in buffering capacity and consequently impair neuronal survival or maturation. Characterizing these pathways would help identify potential targets to prevent or reverse specific anatomic and neurobehavioral alterations following perinatal HO. Using whole hippocampal tissue, we performed an unbiased screen for differentially regulated signaling mediators by GeneChip microarray analysis. At P8, HO caused differential expression of 43 genes (Fig. 6*A*). These genes included Wnt signaling components—*Wnt7b*, *Ctnnb1* (β-catenin), *Dkk3*, *Daam1*—along with genes implicated in neuronal and synapse development, such as *Notch4*, *Syt4* (Synaptotagmin-4), and *Reln* (Reelin), with the latter being expressed in 70% of GAD65-EGFP neurons (Wierenga et al., 2010). We also noted changes in *Gabrb3* (GABA_A_ receptor subunit β3), suggesting a potential dysregulation of the balance between excitatory (E) and inhibitory (I) neurotransmission at P8. Additionally, *Ntrk3* (TrkC) and *Irs2* (insulin receptor substrate 2), a regulator and target of the Akt/mammalian target of rapamycin (mTOR) pathway, respectively (Briaud et al., 2005; Yalvac et al., 2018), are both downregulated, suggesting signaling that may be coregulated with Wnt via GSK3β—a master regulator of hippocampal development processes, such as neurogenesis, apoptosis, and neurite outgrowth (Zhou et al., 2008; Vigneron et al., 2011; Rui et al., 2013; Jurado-Arjona et al., 2016; Suprynowicz et al., 2017). To assess the impact of HO on the Akt-phosphoinositide 3-kinase (PI3K)/GSK3β pathway, we analyzed the phosphorylation levels of different components of the cascade, including upstream and downstream proteins, in whole hippocampal lysates by Western blots. BDNF activates Akt by phosphorylation at S476 through its receptor TrkB. Activated Akt inhibits GSK3β through phosphorylation at S9 to promote cell survival and morphologic differentiation (Beurel et al., 2015). GSK3β activity depends on the balance between the inactivating S9 phosphorylation and the activating phosphorylation at Y216. Following HO, the inhibitory GSK3β phosphorylation at S9 was reduced whereas the Y216 phosphorylation was enhanced, indicative of GSK3β activation (Fig. 6*B*,*C*). Activated GSK3β impedes cell proliferation and exacerbates cell death by inhibiting CREB and β-catenin. Consistent with this mechanism, CREB and β-catenin phosphorylation levels were reduced after HO (Fig. 6*B*,*C*), suggesting that GSK3β plays a role in the cellular alterations induced by perinatal hyperoxia.

**Figure 7.**
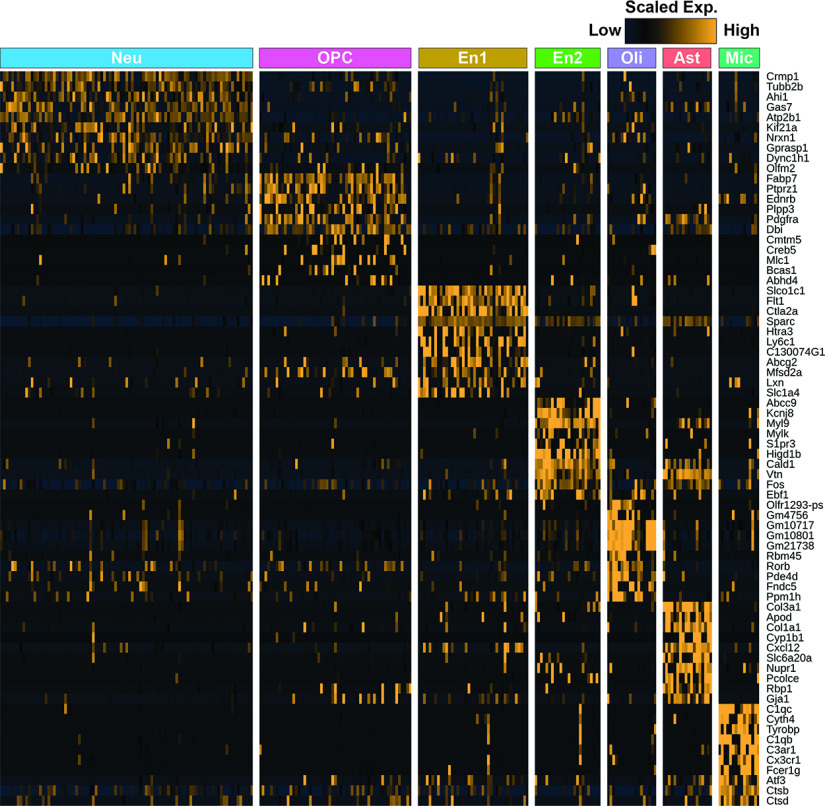
Heatmap of differentially expressed genes in identified cell types from single-cell RNA-seq analysis of P8 mouse hippocampus. The top 10 differentially upregulated genes in each cell type are shown. The row of colored bars at the top of the panels represents the cell types with the same color scheme as in Figure 6*G*. Gene expression levels are scaled across each row as *z* scores, which are represented by heatmap colors.

To further elucidate the molecular mechanism of oxidative stress-mediated neuronal impairment, we performed single-cell RNA-seq of control and HO hippocampi at P8 (Fig. 6*D*). Following cluster analysis, seven distinct clusters of cells were identified and categorized into the following groups: Neuron (Neu), Astrocyte (Ast), Oligodendrocyte progenitor cell (OPC), Oligodendrocyte (Oli), Microglia (Mic), and two distinct endoF7 thelial cell groups (En1 and En2; Figs. 6*E*, 7). In the neuron cluster, consisting of both excitatory and inhibitory neurons, 1289 genes were differentially expressed (1136 upregulated, 153 downregulated; *p* < 0.05) between control and HO conditions, and ingenuity pathway analysis (Qiagen) revealed numerous canonical pathways that were affected in HO hippocampal neurons (Fig. 6*F*). These neuronal pathways included numerous oxidative stress- and mitochondrial dysfunction-related processes, as well as other signaling pathways, including mTOR, IGF-1, and GABA. Inflammation, oxidative phosphorylation, and mitochondrial dysfunction (e.g., *Cox17*, *Ndufa11*; Fig. 6*G*) affect translational responses (e.g., *Rps10*, *Rps11*; Fig. 6*G*), which are events coordinated by mTOR kinase and eukaryotic initiation factor 2 (Samluk et al., 2019). mTOR, growth factor, and neuronal survival pathways mTOR/Akt/IGF-1 (e.g., *Igfbp7*, *Mapkap1*, *Mras*, *Rpsa*; Fig. 6*G*) are regulated by GSK3β (Urbanska et al., 2018). These data support the interpretation that changes in GSK3β-mediated signaling in response to oxidative stress observed in GeneChip microarray and Western blotting occur in neurons. To determine whether GSK3β can be activated in newly born granule cells and interneurons after HO, the phosphorylation status of GSK3β was assessed in POMC-EGFP and GAD65-GFP neurons in P8 *ex vivo* slices acutely exposed to high oxygen for 20 min. As expected, p(Y216)-GSK3β was expressed in POMC-expressing granule cells of the DG and in GAD65-expressing neurons of the CA1 (Fig. 6*H*).

### Pharmacological GSK3β inhibition prevents HO-induced alterations in progenitor cell and neuronal development

**Figure 8.**
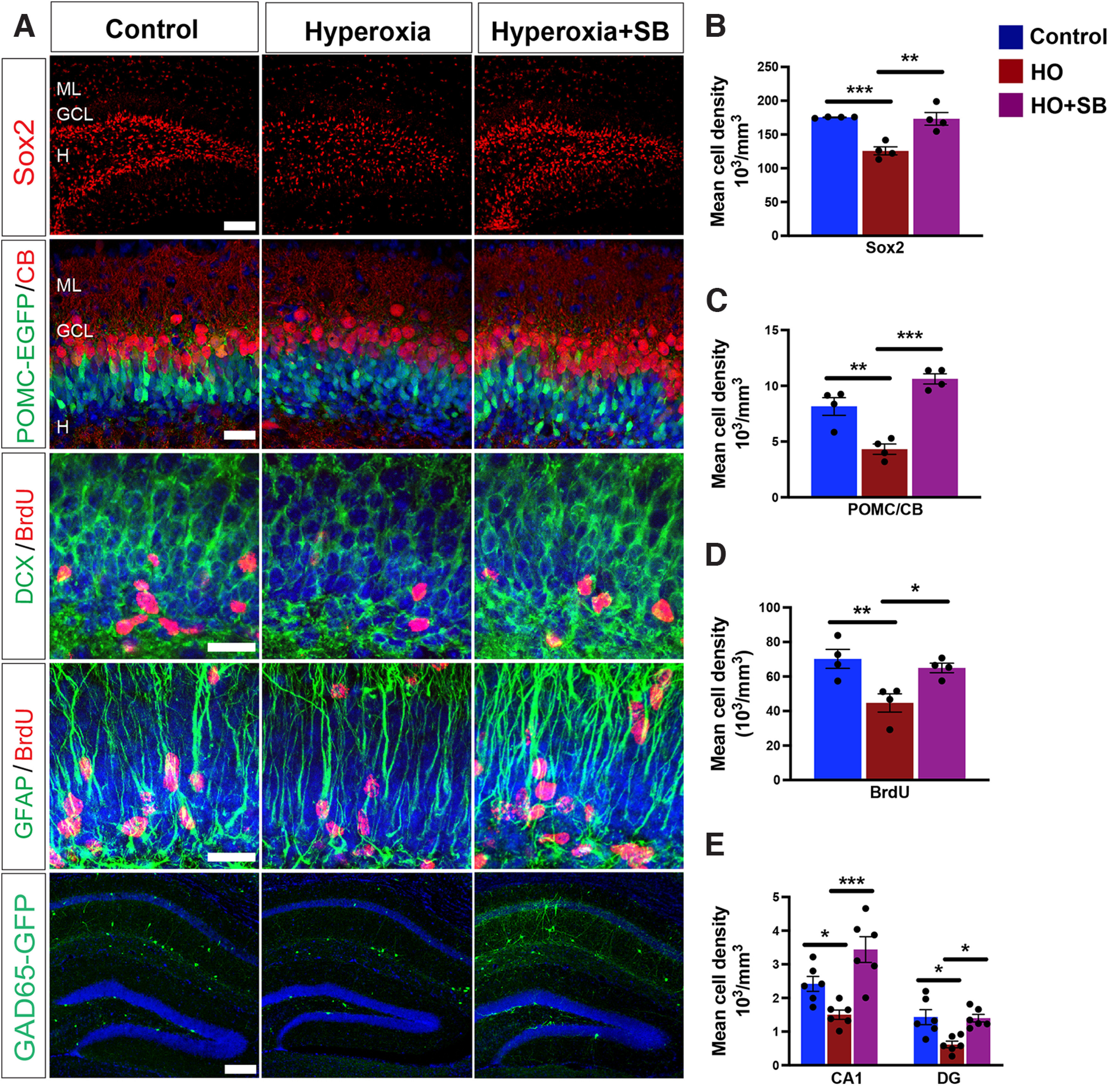
Inhibition of GSK3β activity prevents HO-induced deficits in progenitor cell proliferation and neuronal development. ***A***, Representative confocal images of Sox2^+^ dentate progenitor cells (red), POMC-EGFP^+^ (green) and CB^+^ (red) colabeled cells, DCX+ (green) and BrdU^+^ (red) colabeled cells, GFAP+ (green) and BrdU^+^ (red) colabeled cells, and GAD65-GFP^+^ interneurons (green) in hippocampi. ML, Molecular layer; GCL, granule cell layer; H, hilus. ***B–D***, Quantification of POMC-EGFP^+^/CB^+^ (***B***), BrdU^+^ (***C***), or Sox2^+^ (***D***) cells in the P8 DG. ***E***, Quantification of GAD65-GFP^+^ cells in CA1 and DG at P60. Student's *t* test: *p < 0.05; **p < 0.01; ***p < 0.005. Scale bars: ***A***, Sox2, GAD65-GFP, 100 µm; ***A***, POMC-EGFP/CB, 50 µm; ***A***, DCX/BrdU, GFAP/BrdU, 25 µm.

To determine the role of GSK3β in HO-mediated reduction in cell proliferation and development, we administered SB216763 (SB), a GSK3β-specific inhibitor, before hyperoxia exposure at P6 and analyzed progenitor cell development and GAD65-EGFP neurons. The decline in the number of Sox2 progenitor cells at P8 in the DG following HO was prevented by SB (Fig. 8*A*,*B*). Newly generated cells in the DG transition from immature granule cells that express POMC or DCX to calretinin- and calbindin (CB)-expressing cells before integrating into existing networks. We analyzed the developmental transition of newly generated cells in the DG at P8 and found that SB pretreatment reversed the HO-induced decrease in POMC/CB-colabeled cells (Fig. 8*A*,*C*). Additionally, the reduction in BrdU^+^ proliferating cells in the DG following HO was also prevented by SB pretreatment (Fig. 8*A*,*D*). BrdU was predominantly expressed in DCX^+^ and GFAP^+^ cells indicative of ongoing neurogenesis and gliogenesis (Fig. 8*A*). Indeed, at P60, HO significantly decreased the number of hippocampal GAD65-GFP neurons in CA1 and DG, whereas no decrease in GAD65-GFP neurons was observed after SB pretreatment (Fig. 8*A*,*E*). Overall, these results show that pharmacological inhibition of GSK3β before the hyperoxic injury prevents the HO-induced dysmaturation of neuronal progenitors and restores interneuron development in the hippocampus.

### Pharmacological GSK3β inhibition prevents HO-induced alterations to inhibitory neurotransmission

**Figure 9.**
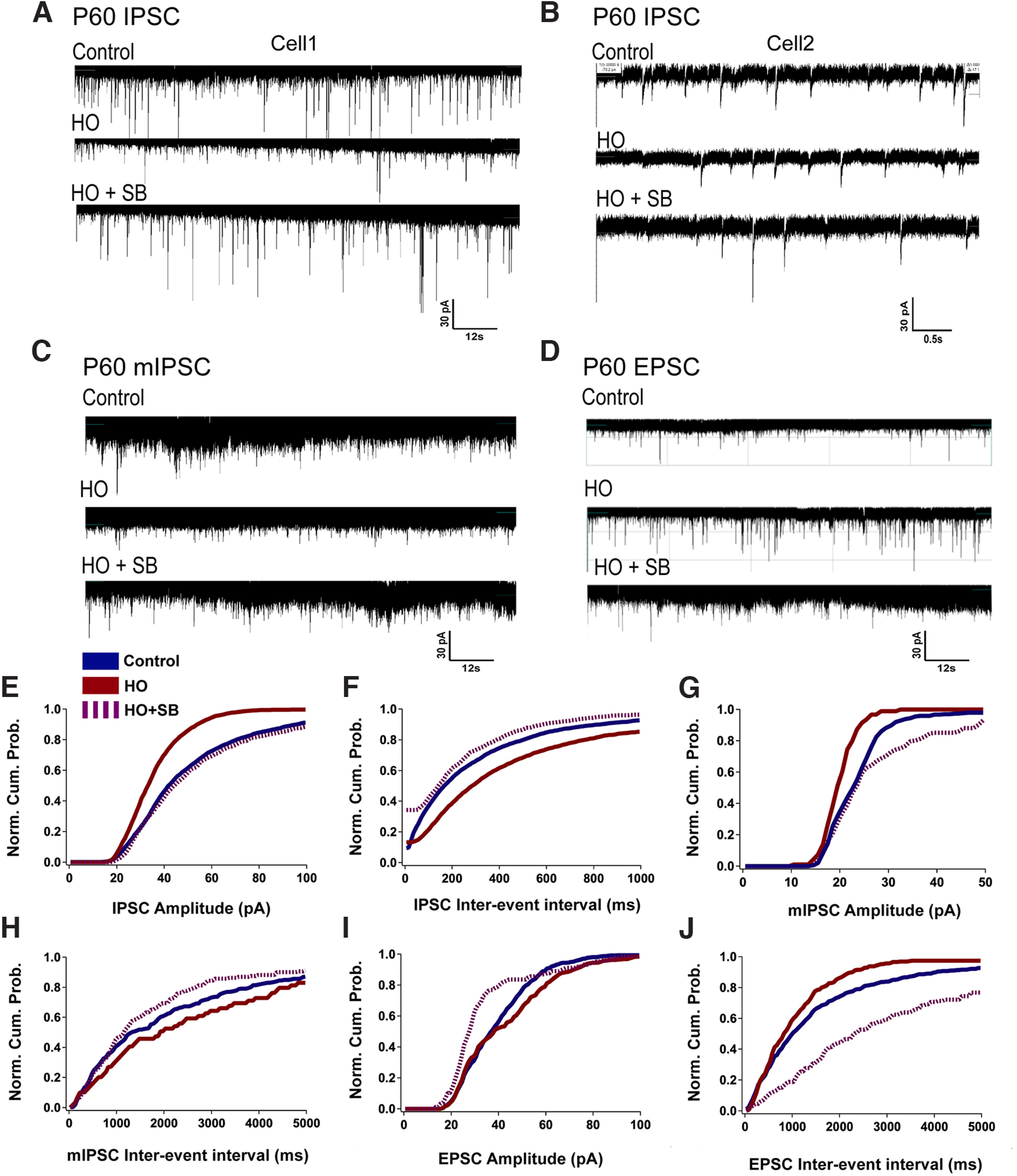
GSK3β inhibition reverses HO-induced imbalance of excitation and inhibition in the P60 hippocampus. ***A***, ***B***, Spontaneous IPSC traces of two independent cells per condition showing the effect of HO (middle) and pretreatment with SB (HO + SB, bottom). ***C***, Representative traces of mIPSC from control (top), HO (middle), and HO + SB neurons (bottom). ***D***, Representative traces of EPSC from control (top), HO (middle), and HO + SB neurons (bottom). ***E–J***, Normalized cumulative probability plots showing control (blue), HO (red), and HO + SB (dashed magenta) changes in IPSC amplitude (***E***), IPSC interevent interval as an indicator of frequency (***F***), mIPSC amplitude (***G***), mIPSC interevent interval (***H***), EPSC amplitude (***I***), and EPSC interevent interval (***J***).

As the effects of SB are likely to impact hippocampal function, we determined the physiological consequence of pharmacological GSK3β inhibition on inhibitory neurotransmission in the CA1 with whole-cell patch-clamp recordings at P60. We first analyzed spontaneous IPSCs that were pharmacologically isolated with a cocktail of CNQX (10 µm), SCH50911 (20 µm), and APV = DL-2-amino-5-phosphonovaleric acid (50 µm) at a holding potential of −70 mV. The amplitude and frequency of IPSCs were significantly reduced in HO-exposed mice (age, P60; Fig. 9*A*,*B*,*E*,*F*). However, pretreatment with SB restored both the amplitude and frequency of IPSCs to control levels (Fig. 9*A*,*B*,*E*,*F*). Similarly, the amplitude and frequency of miniature IPSCs (mIPSCs) were significantly reduced after HO exposure and reversed in SB-treated mice (Fig. 9*C*,*G*,*H*). On the other hand, HO led to increased amplitude and frequency of EPSCs in the CA1, which was reversed by SB (Fig. 9*D*,*I*,*J*). These data demonstrate that pharmacological inhibition of GSK3β is sufficient to reverse the imbalance between synaptic excitation and inhibition caused by perinatal HO and further supports the role of GSK3β signaling in the HO-induced dysmaturation of the hippocampus.

### GSK3β ablation in hippocampal interneurons reverses HO-induced alterations to inhibitory neurotransmission and cognitive deficits

**Figure 10.**
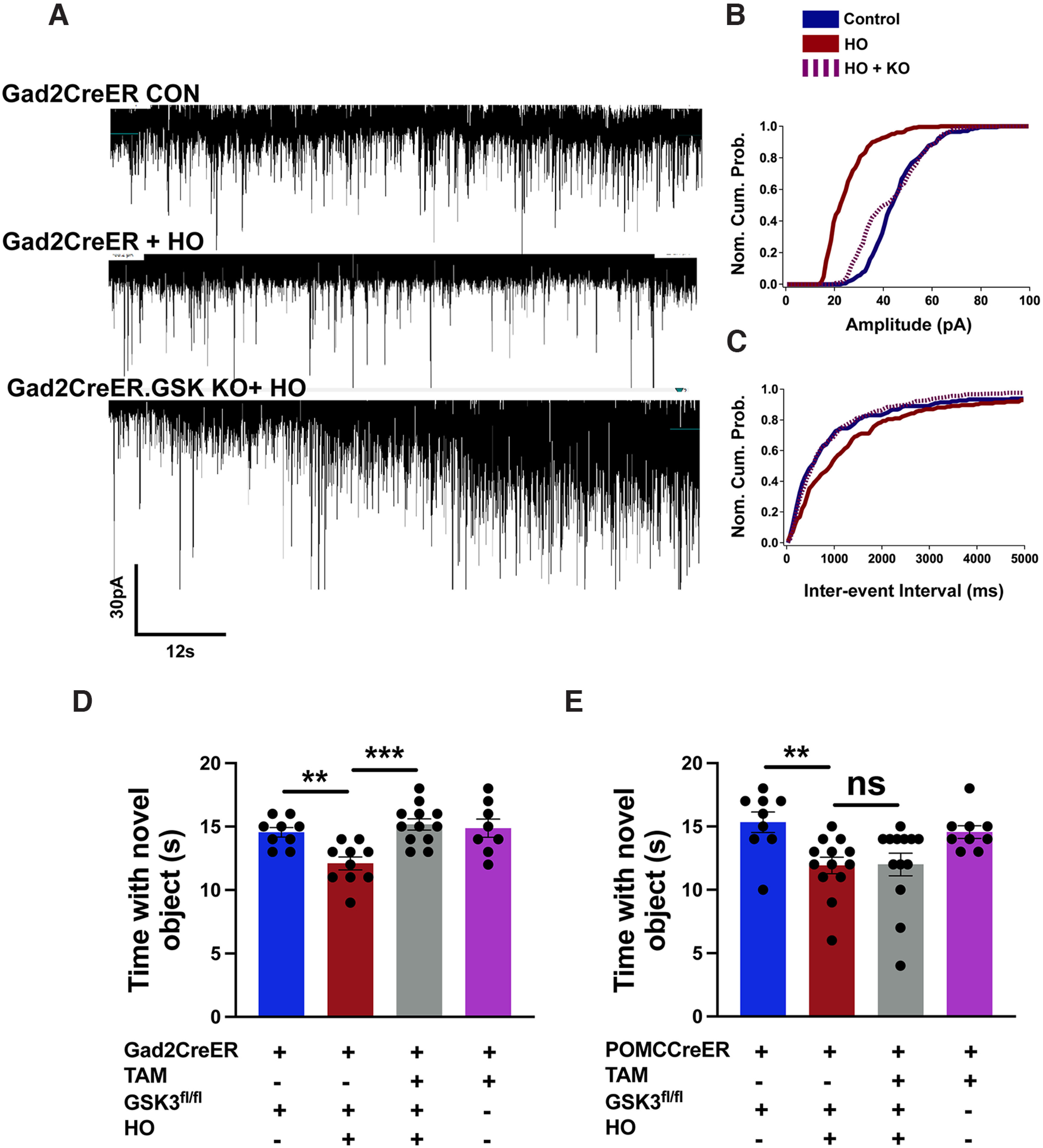
Conditional deletion of GSK3β in Gad2-expressing cells, not POMC-expressing cells, improves hippocampus-dependent learning deficits in mice challenged with oxidative stress. ***A***, Representative traces of spontaneous IPSCs from control (top), HO (middle), and HO + Gad2CreER^T2^-GSK3β^flox/flox^ (bottom) neurons. ***B***, ***C***, Normalized cumulative probability plots showing control (blue), HO (red), and HO + Gad2CreER^T2^-GSK3β^flox/flox^ (dashed magenta) changes in IPSC amplitude (***B***) and IPSC interevent interval (***C***). ***D***, In the novel object recognition test, ablation of GSK3β in Gad2CreER^T2^ neurons preserves learning and memory abilities. ***E***, In the same test, the ablation of GSK3β in POMC-CreER^T2^-expressing cells fails to improve the learning deficit caused by HO. NS, Not significant; TAM, tamoxifen. One-way ANOVA with Tukey's *post hoc* test: *p < 0.05, **p < 0.005, ***p < 0.001.

To investigate the role of GSK3β specifically in GAD65 interneurons and in newly generated POMC-expressing cells of the DG, we generated two conditional knock-out (KO) mouse lines by breeding *GAD2CreER*^T2^*.GCAMP5TdTomato*^+^ mice with GSK3β^flox/flox^ or *POMCCre*ER^T2^.*GCAMP5TdTomato*^+^ with GSK3β^flox/flox^ mice. To knock down GSK3β expression in these individual mouse lines, tamoxifen was injected at P4, 48 h before HO exposure. Similar to findings using SB, whole-cell patch-clamp recordings (Fig. 10*A*) demonstrate that the ablation of GSK3β in Gad2-expressing cells reverses the HO-induced reduction in IPSC amplitude and frequency at P60 (Fig. 10*B*,*C*). To evaluate the behavioral effects of GSK3β KO in POMC- and Gad2-expressing cells, we assessed recognition memory using the NORT at P60. We confirmed that HO significantly reduced the time spent with the novel object when tamoxifen was not administered to *GAD2CreER* or *POMCCreER*-GSK3β^flox/flox^ mice (Fig. 10*D*,*E*; but see also Fig. 4). Following the ablation of *GSK3*β in Gad2-expressing cells, mice recovering from HO spent a significantly longer time with novel objects, indicative of a normal behavioral phenotype (Fig. 10*D*). However, the ablation of *GSK3*β in POMC-expressing cells did not reverse the HO-induced impairment in recognition memory (Fig. 10*E*). Together, these results indicate that aberrant activation of GSK3β in Gad65-expressing interneurons, rather than in POMC-expressing newly born granule cells, plays a significant role in mediating the HO-induced deficit in hippocampal-dependent learning tasks.

## Discussion

Premature birth increases the risk for oxidative stress and its associated tissue injuries. As the hippocampus undergoes continuous postnatal development, it remains particularly vulnerable to damage from oxidative stress. To overwhelm antioxidant defenses with oxygen in our model, we used a high level of ambient oxygen (80–85%) for a short time period (48 h) in normally delivered mouse pups, allowing for an investigation into mechanisms underlying neonatal brain injury. In this study, we demonstrate that two cell types—newly generated cells that are POMC^+^ and a subset of interneurons that express GAD65—were vulnerable to HO-induced oxidative stress. Previous work has demonstrated the critical roles of proper postnatal cell proliferation and interneuron function in hippocampal-dependent cognitive function (van Praag et al., 2002).

There is substantial evidence indicating that the rate of postnatal cell proliferation and developmental transition of neuroblasts in the subgranular zone correlates with hippocampus-dependent activities such as learning and cognition (Gould et al., 1999). Consequently, changes in cell proliferation caused by intrinsic signaling or environmental cues (Kempermann et al., 1997; Bruel-Jungerman et al., 2005; Tashiro et al., 2007) impair hippocampus-dependent behaviors (Ambrée et al., 2014; Naninck et al., 2015). Consistent with previous reports in neonatal rats where HO resulted in reduced cell number and DG volume (Porzionato et al., 2015), our findings demonstrate that HO diminishes the proliferative capacity of the DG, as indicated by the decreased number of Sox2^+^ (stem/progenitor) cells and cells that incorporate BrdU. Sox2-expressing cells undergo symmetric cell division for clonal expansion and asymmetric division to generate postmitotic neuroblasts. Our observation that caspase-3 activity is increased in parallel with reduced mitotic cell-cycle activity suggests that both increased cell death as well as compromised intrinsic regenerative potential may underlie aberrant changes in hippocampal plasticity. It has been suggested that memory coding in the hippocampus relies on continuous hippocampal neurogenesis, whereby newly generated neuroblasts undergo a developmental transition from calretinin^+^ to CB^+^ cells as they migrate to and integrate into existing networks (Deng et al., 2010). In our study, exposure to HO reduces the density of CB^+^ cells, indicating that normal neuroblast maturation is impaired. These observations are consistent with the contribution of newly generated CB^+^ neurons in HO-affected memory behaviors, given a reported association between reduced hippocampal CB and spatial memory deficits following neonatal hypoxia-ischemia (Goffigan-Holmes et al., 2018).

The developing brain suffers white matter damage and GABAergic neuron loss in prematurely born infants (Robinson et al., 2006). In addition, the functional integrity of the hippocampus depends on an appropriate balance between excitatory (i.e., glutamatergic principal cells) and inhibitory (i.e., GABAergic interneurons) neurotransmission (E/I balance). Thus, the loss of interneurons that modulate the basal activity of the hippocampus alters the E/I balance, resulting in hippocampal dysfunction and abnormal behavior in a number of diseases (Benes, 1998; Konradi et al., 2011; Schobel et al., 2013; Caletti et al., 2015; Reichel et al., 2015). In our study, exposure to HO leads to morphologic dysmaturation and loss of interneurons across the hippocampus. Furthermore, *in vitro* electrophysiological analysis using whole-cell patch-clamp recording demonstrated altered intrinsic physiological properties of HO-exposed Gad2^+^ cells, which is consistent with *in vivo* optogenetic analysis that demonstrated decreased basal activity of these cells. Consequently, both IPSCs and mIPSCs were reduced, indicating a decline in overall inhibitory tone in the hippocampus in the presence or absence of action potential-mediated events. On the other hand, the EPSCs were enhanced, suggesting that the E/I balance is shifted toward excitation, giving rise to hyperactive circuits. The disruption of the E/I balance because of GABAergic dysfunction in our model parallels clinical observations in children born preterm (Lacaille et al., 2019). Interestingly, the postinjury optogenetic stimulation of Gad2-expressing neurons demonstrated the reversibility of the impairment, such that the selective optogenetic and chemogenetic activation of Gad2 interneurons strikingly led to improved performance in the novel object recognition test.

Using microarray and single-cell sequencing-based analyses, we identified antioxidant pathways, NMDA and GABA receptor signaling, and GSK3β as dysregulated mechanisms underlying aberrant cellular development and behavioral anomalies because of HO. This is in agreement with previous reports that demonstrated an association between oxidative stress-induced neurodegeneration and GSK3β (Wang et al., 2013). In particular, following exposure to HO, we observed reduced expression of BDNF and its receptor TrkB, along with Wnt, Akt, and PI3K signaling components. Under normal conditions, activated Akt regulates GSK3β to control the balance between activated and inhibitory forms of the enzyme, ensuring appropriate functions of the enzyme while preventing pathologic actions related to hyperactivation (Beurel et al., 2015). Our study demonstrates that HO-induced oxidative stress impairs the activity of the Akt/PI3k pathway, removing the repression of GSK3β activity, and shifting the balance between activated and inhibitory forms of the enzyme in favor of activation. Overactivation of GSK3β promotes cell death, impairs cell proliferation and maturation of newly born granule cells, as well as the development and function of interneurons (Matsuda et al., 2019). Our finding that GSK3β inhibition prevented these cellular damages, ultimately restoring the E/I balance, not only contributes to the understanding of the mechanism of HO-induced hippocampal dysmaturation, but also opens up the possibility of using agents targeting the Akt/GSK3β pathway to prevent it.

As the primary subcortical structure for memory formation, the hippocampus, through reciprocal connections with the neocortex and other subcortical structures (O'Donnell and Grace, 1995; Pikkarainen et al., 1999), governs cognitive function and influences many other behaviors (Sahay and Hen, 2008). Hippocampal plasticity, aided by continuous neurogenesis, is crucial for its function. Any reduction in the rate of postnatal cell proliferation in the DG will impair hippocampus-dependent function (Ambrée et al., 2014; Naninck et al., 2015). Memory formation in the hippocampus is accompanied by synaptic plasticity at inhibitory interneurons (Ruediger et al., 2011; Donato et al., 2013, 2015) and is dependent on activity levels within the hippocampus (Heckers and Konradi, 2015). Thus, the loss of inhibitory interneurons that is associated with altered E/I balance would impair hippocampus-dependent learning. In fact, interneuron precursor transplants in a mouse model of hippocampal disinhibition were reported to reverse psychosis (Gilani et al., 2014; Reichel et al., 2015).

Our results indicate that altered hippocampal neurogenesis and GABAergic dysfunction underlie learning deficits in HO mice. Importantly, the *in vivo* stimulation of hippocampal interneurons in HO animals indicates that (1) GABAergic inadequacy plays a prominent role in the cognitive pathophysiology of HO, and (2) increasing interneuron function specifically in the postinjury hippocampus corrects the learning deficit caused by HO. Using an inducible gene-targeted approach to reduce GSK3β levels in POMC-expressing cells or Gad2-expressing interneurons, we showed that modulating the levels of GSK3β in interneurons, but not in POMC-expressing cells, significantly improved inhibitory neurotransmission and reversed memory deficits because of HO. This finding indicates that GSK3β activity in the loss and dysmaturation of interneurons plays a critical role in the behavioral pathology of HO. The differences in behavioral outcomes between cell-specific GSK3β ablation models may reflect that postnatal cell regeneration in the hippocampus involves multiple cell lineages including Sox2^+^ and Dcx^+^ cells; thus, targeting a subpopulation of immature neuroblasts may not adequately protect against the damaging effect of HO on hippocampal remodeling and function.

In summary, our study demonstrates that E/I imbalance and learning deficits arising from oxidative stress-induced neonatal brain injury are primarily mediated through dysmaturation and loss of interneurons in the hippocampus. These findings also identified dysregulation of GSK3β as a molecular target and revealed potential preventative and postinjury interventions to ameliorate cognitive impairment in oxidative stress-mediated developmental brain injury.
